# Trends in Emergency Department Visits Involving Cannabis Hyperemesis Syndrome Identified Using a New Diagnosis Code — United States, January 2023–May 2026

**DOI:** 10.15585/mmwr.mm7530a2

**Published:** 2026-08-06

**Authors:** Alana M. Vivolo-Kantor, Stephen Liu, Lauren J. Tanz, Christine L. Mattson, Josh Schier

**Affiliations:** 1 Division of Overdose Prevention, National Center for Injury Prevention and Control, CDC.

**Summary**

**What is already known about this topic?**

The prevalence of cannabis hyperemesis syndrome (CHS), a condition characterized by cyclical nausea and vomiting associated with frequent cannabis use, increased sharply in the United States during 2016–2022.

**What is added by this report?**

The proportion of all-cause emergency department visits that involved CHS were higher after implementation of a new *International Classification of Diseases, Tenth Revision, Clinical Modification* diagnosis code, suggesting improved recognition and previous underestimation of the public health impact of CHS. Higher proportions were observed among younger persons and females.

**What are the implications for public health practice?**

The new CHS-specific diagnosis code has improved CHS surveillance and estimation of public health impact. However, increased clinical recognition and education about risks of frequent cannabis use are still needed, particularly in younger populations.

## Abstract

Cannabis hyperemesis syndrome (CHS) is a condition characterized by cyclical nausea and vomiting and is associated with frequent cannabis use. Data from CDC’s National Syndromic Surveillance Program were analyzed to examine trends in CHS-involved emergency department (ED) visits before and after implementation of a new, CHS-specific *International Classification of Diseases, Tenth Revision, Clinical Modification* diagnosis code on October 1, 2025. Monthly CHS-involved ED visits per 10,000 all-cause visits were assessed overall and by demographic characteristics. During January 2023–May 2026, a total of 199,565 ED visits involved CHS. During January 2023–September 2025, the proportion of ED visits that involved CHS remained mostly steady. In the first month after implementation of the new diagnostic code, the proportion of CHS-involved ED visits increased from 3.35 per 10,000 ED visits in September 2025 (preimplementation) to 11.26 per 10,000 ED visits in October 2025 (postimplementation). During the first 8 months after implementation of the new code (October 2025–May 2026), average monthly proportions of CHS-involved ED visits were 3.7 times as high as the monthly average during January 2023–September 2025. Higher proportions of CHS-involved ED visits were observed among persons aged 15–24 years and females, with more pronounced impacts among some demographic groups after code implementation. The abrupt, sustained increase might partly reflect improved recognition and coding of CHS rather than a true rise in incidence. These findings highlight a likely underestimated impact of CHS, suggesting the need to strengthen education about the risks associated with frequent cannabis use, expand ongoing surveillance efforts, and improve clinical recognition of CHS through continuing medical education and implementation of routine cannabis use assessment in ED settings.

## Introduction

Cannabis hyperemesis syndrome (CHS) is a syndrome attributed to prolonged, frequent cannabis use, characterized by sudden episodes of severe nausea, vomiting, and abdominal pain (*1*). Reports of suspected CHS increased sharply during 2016–2022, with the largest increase occurring during the COVID-19 pandemic (*2*); the increase also coincided with widespread state-level cannabis legalization in the United States, although cannabis use remains illegal under federal law (*3*). Persons with CHS often seek medical care because symptoms can be severe and debilitating, with outcomes ranging from symptom resolution after cessation of cannabis use to, in rare cases, death (*1*).

Trends in cannabis use might be contributing to the increasing prevalence and recognition of CHS. The prevalence of past-month and daily or near daily cannabis use has been increasing, particularly among young adults and females (*4*). In addition, concentrations of tetrahydrocannabinol (THC), the primary psychoactive compound in cannabis, have increased in cannabis products over time (*5*). Higher THC concentration (>10%) is associated with continued and more frequent cannabis use among adolescents and young adults (*6*). Although THC concentration has not been directly linked to CHS, higher-potency products are associated with more frequent use, the primary risk factor for CHS (*1*,*6*).

CHS is likely underrecognized in both adolescent and adult populations. Clinicians might not routinely assess or document patients’ cannabis use; CHS can be misdiagnosed as other gastrointestinal conditions, such as cyclical vomiting syndrome (*7*). To improve clinical identification, a specific *International Classification of Diseases, Tenth Revision, Clinical Modification (ICD-10-CM)* discharge diagnosis code for CHS was introduced on October 1, 2025. This report examines trends in CHS-involved emergency department (ED) visits before and after implementation of this code.

## Methods

### Data Source

CHS-involved ED visits were identified using data from CDC’s National Syndromic Surveillance Program (NSSP), a collaboration with state and local health departments, CDC, and other partners to provide near real-time electronic health record data. Approximately 7,500 health care facilities covering 50 states, the District of Columbia, and Guam contribute data to NSSP, representing 83% of U.S. EDs. Analyses were limited to 3,636 (70.0%) of 5,190 ED facilities consistently reporting data for January 1, 2023–May 31, 2026.*

### Syndrome Definition and Data Classification

On October 1, 2025, a new *ICD-10-CM* diagnosis code for CHS (R11.16) was introduced. For ED visits that occurred before introduction of the new code (January 2023–September 2025) CHS was identified using a definition consisting of a combination of codes for cyclical vomiting (R11.15) or nonspecific emesis (R11) in tandem with a code for a cannabis-related diagnosis (F12x or T40.7Xx), as developed and assessed for clinical sensitivity (*2*). For ED visits during October 2025–May 2026, CHS-involved visits were identified using the newly implemented R11.16 code; visits were also included if they met the previous definition (*2*) but did not include the new R11.16 code.

### Data Analysis

Monthly counts per 10,000 all-cause ED visits are analyzed overall and by age group (11–14, 15–24, 25–34, 35–44, and ≥45 years), sex (male or female), and race and ethnicity for the entire study period using the two different syndrome definitions. Persons of Hispanic or Latino (Hispanic) origin might be of any race but are categorized as Hispanic; all racial groups are non-Hispanic.^†^ Data for children aged <11 years are not reported because of low case count and suppression rules (i.e., fewer than 20 cases per month). During October 2025–May 2026, monthly counts and proportions^§^ were aggregated and reported overall and by age group, sex, and race and ethnicity. This activity was reviewed by CDC, deemed not research, and conducted consistent with applicable federal law and CDC policy.^¶^

## Results

**FIGURE 1:**
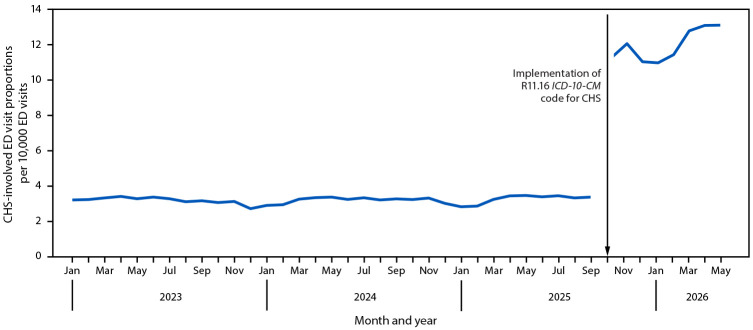
Monthly proportions of emergency department visits involving cannabis hyperemesis syndrome* — National Syndromic Surveillance Program, United States, January 2023–May 2026 **Abbreviations: **CHS = cannabis hyperemesis syndrome; ED = emergency department; *ICD-10-CM* = *International Classification of Diseases, Tenth Revision, Clinical Modification*. * During January 1, 2023–September 30, 2025, the CHS case definition included a combination of discharge diagnosis codes for cyclical vomiting (R11.15) or nonspecific emesis (R11) in tandem with codes for cannabis-related diagnosis (F12x or T40.7Xx). During October 1, 2025–May 31. 2026, the new *ICD-10-CM* diagnosis code for CHS (R11.16) was used to identify CHS-involved visits. During this period, visits were also included if they met the definition used during January 1, 2023–September 30, 2025, but did not include the new R11.16 code.

During January 2023–May 2026, a total of 199,565 CHS-involved ED visits were identified among 407,326,613 all-cause ED visits. Using a previous definition (*2*), overall proportions of CHS-involved ED visits were 3.19 per 10,000 ED visits in January 2023 and 3.35 in September 2025 (Figure 1), ranging from 2.69 in December 2023 to 3.44 in May 2025. After the new diagnosis code was introduced, the proportion of visits identified increased substantially (from 3.35 per 10,000 ED visits in September 2025 to 11.26 in October 2025). During this period, notable fluctuations occurred in average monthly proportions of CHS-involved ED visits, ranging from 12.05 in November 2025, to 10.96 in January 2026, to 13.10 in May 2026 (Figure 1). During October 2025–May 2026, the average monthly proportion of CHS-involved ED visits was 11.97 (Table), 3.7 times as high as the monthly average (3.19) during January 2023–September 2025.

**TABLE:** Total number and average monthly proportions* of emergency department visits involving cannabis hyperemesis syndrome, by age, sex, and race and ethnicity — National Syndromic Surveillance Program, United States, October 2025–May 2026^†^

Characteristic	Total no. of visits for any cause	Average no. of CHS-involved ED visits per month	Average CHS-involved ED visit proportions per month
**Total**	**94,950**	**11,868.75**	**11.97**
**Age group, yrs (n = 93,947)^§^**			
11–14	**711**	88.88	3.19
15–24	**34,374**	4,296.75	38.70
25–34	**29,653**	3,706.63	28.28
35–44	**15,885**	1,985.63	15.89
≥45	**13,324**	1,665.50	3.49
**Sex (n = 94,853)^¶^**			
Female	**55,258**	6,907.25	12.65
Male	**39,595**	4,949.38	11.13
Race and ethnicity (n = 92,080)**			
AI/AN	**902**	112.75	15.14
Asian and NH/PI	**989**	123.63	5.32
Black or African American	**27,000**	3,375	17.60
White	**43,281**	5,410.13	10.47
Hispanic or Latino	**16,653**	2,081.63	12.08
Multiple or other races	**3,255**	407	9.59

**Abbreviations:** AI/AN = American Indian or Alaska Native; CHS = cannabis hyperemesis syndrome; ED = emergency department; NH/PI = Native Hawaiian or Pacific Islander.

* Proportions of CHS-involved ED visits were calculated by summing the total number of visits meeting the case definition, dividing by the total number of ED visits for any cause, and scaling to 10,000 ED visits.

^†^ During January 1, 2023–September 30, 2025, the CHS case definition included a combination of discharge diagnosis codes for cyclical vomiting (R11.15) or nonspecific emesis (R11) in tandem with codes for cannabis-related diagnosis (F12x or T40.7Xx). During October 1, 2025–May 31, 2026, the new *International Classification of Diseases, Tenth Revision, Clinical Modification* diagnosis code for CHS (R11.16) was used to identify CHS-involved visits. During this period, visits were also included if they met the definition used during January 1, 2023–September 30, 2025, but did not include the new R11.16 code (14,988).

^§^ Children aged 0–10 years were excluded from analysis because of low case count and suppression rules (i.e., fewer than 20 cases per month). Sample sizes (the number of total ED visits for any cause) by age group were as follows: 11–14 years, 2,225,666; 15–24 years, 8,882,394; 25–34 years, 10,485,979; 35–44 years, 9,995,358; and ≥45 years, 38,160,064.

^¶^ Sample sizes (the number of total ED visits for any cause) were 43,691,253 for females and 35,561,303 for males.

** Persons of Hispanic or Latino (Hispanic) origin might be of any race but are categorized as Hispanic; all racial groups are non-Hispanic. Sample sizes (the number of total ED visits for any cause) by race and ethnicity were as follows: AI/AN, 595,866; Asian and NH/PI, 1,857,387; Black or African American, 15,338,697; White, 41,341,916; Hispanic, 13,790,061; and multiple or other races, 3,394,679.

**FIGURE 2:**
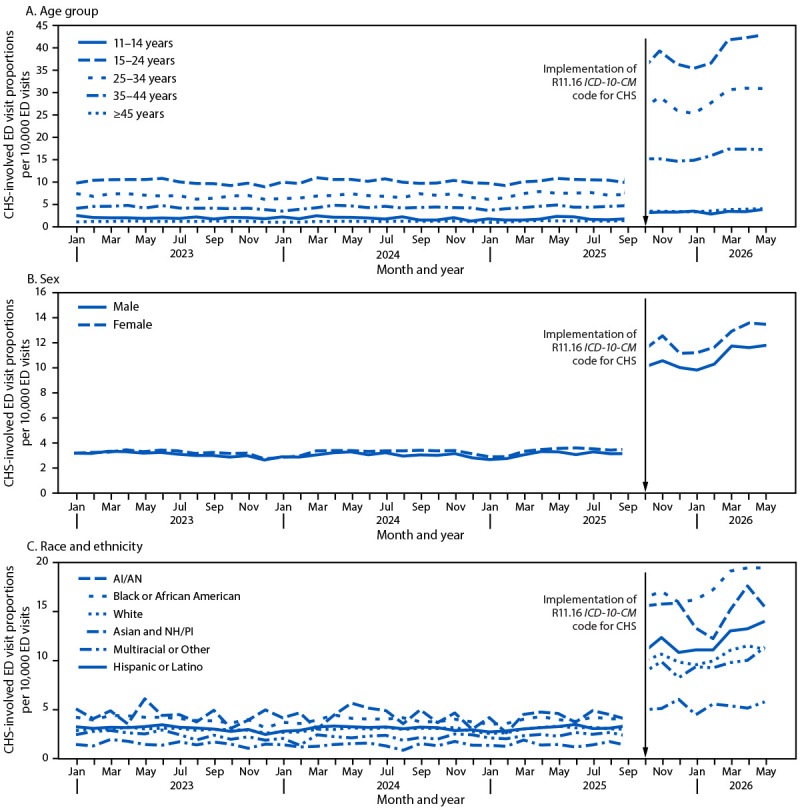
Monthly proportions of emergency department visits involving cannabis hyperemesis syndrome,* by age (A), sex (B), and race and ethnicity (C)^†^ — National Syndromic Surveillance Program, United States, January 2023–May 2026 **Abbreviations:** AI/AN = American Indian or Alaska Native; CHS = cannabis hyperemesis syndrome; ED = emergency department; *ICD-10-CM* = *International Classification of Diseases, Tenth Revision, Clinical Modification*; NH/PI = Native Hawaiian or Pacific Islander. * Visits with missing or unknown values for certain variables were excluded from analyses of that variable. A minimal amount of data was missing for each of the demographic variables; age (0.45%), sex (0.22%), and race and ethnicity (5.20%). During January 1, 2023–September 30, 2025, the CHS case definition included a combination of discharge diagnosis codes for cyclical vomiting (R11.15) or nonspecific emesis (R11) in tandem with codes for cannabis-related diagnosis (F12x or T40.7Xx). During October 1, 2025–May 31, 2026, the new *ICD-10-CM* diagnosis code for CHS (R11.16) was used to identify CHS-involved visits. During this period, visits were also included if they met the definition used during January 1, 2023–September 30, 2025, but did not include the new R11.16 code. ^†^ Persons of Hispanic or Latino (Hispanic) origin might be of any race but are categorized as Hispanic; all racial groups are non-Hispanic.

Before implementation of the new code in October 2025, temporal trends of CHS-involved ED visits per 10,000 ED visits by age group, sex, and race and ethnicity also remained steady during January 2023–September 2025 (Figure 2). During this period, the highest average proportion of monthly CHS-involved ED visits per 10,000 ED visits was identified among persons aged 15–24 years (9.99), females (3.28), and American Indian or Alaska Native persons (4.33). After the new CHS code was implemented, a few differences emerged. Although the period of observations is limited to 8 months, the data suggest a potential increase in average monthly CHS-involved ED visits for persons aged 15–24 and 25–34 years and those who are Hispanic, Black or African American (Black), White, and of multiple or other races. Throughout this period, higher average proportions of monthly CHS-involved ED visits per 10,000 ED visits were identified among persons aged 15–24 years (38.70), Black persons (17.60), and females (12.65) (Table).

## Discussion

The proportion of CHS-involved ED visits remained steady during January 2023–September 2025 until the introduction of a new diagnosis code for CHS in October 2025, after which a marked increase occurred. The abruptness and sustained magnitude of the increase likely reflects, at least in part, improved clinical recognition and coding of CHS rather than a true and immediate rise in incidence. The stability of trends before October 2025, including those across demographic subgroups, further supports the notion that the observed shift is likely driven by changes in coding practices. However, the higher levels after implementation of the new code might represent more accurate estimates of CHS impact in ED settings. These findings underscore the importance of accounting for coding changes when interpreting temporal trends and highlight the potential use of ED data for monitoring CHS and related substance use patterns.

Proportions of CHS-involved ED visits were high in younger age groups, particularly those aged 15–24 years. CHS typically occurs among those with heavy, frequent, and long-term cannabis use (*1*); previous studies have characterized CHS as a condition associated with heavy cannabis use over extended periods (*8*). However, emerging evidence indicates that symptom onset can occur much sooner than was previously estimated, including within the first year of cannabis use (*8*). Some studies have reported delays of several years from symptom onset to diagnosis (*8*), suggesting that CHS might be underrecognized, particularly early in its course. Although data remain limited, high proportions of CHS-involved ED visits among younger age groups might reflect changing patterns of cannabis use behaviors, including earlier age at initiation, increased use of high-potency THC products, evolving methods of use (e.g., vaping or dabbing**), changing perceptions of risk, or increased frequency of use among younger populations (*9*).

The introduction of a specific diagnosis code for CHS in October 2025 represents an important advancement for public health surveillance. Before its implementation, many CHS cases were likely not identified because of frequent misclassification as cyclical vomiting syndrome and reliance on secondary cannabis-related codes (*2*,*7*). The new code enables more consistent documentation and might facilitate clinical recognition and direct case ascertainment, improving the ability to monitor the public health impact of CHS, detect changes in incidence, and understand risk factors across populations.

### Limitations

The findings in this report are subject to at least five limitations. First, the *ICD-10-CM* codes used to identify CHS before October 1, 2025, were nonspecific and imprecise, which might have resulted in misclassification. Even after implementation of the new diagnosis code, CHS-involved ED visits might be underreported or misclassified because identification relies on clinician recognition and documentation. Second, these data only include patients with symptoms severe enough to seek emergency care and therefore likely underestimate the true incidence of CHS. Third, ascertainment of CHS depends, in part, on patient self-report of cannabis use, which might be affected by social desirability or recall bias. Fourth, despite relatively steady ED visit numbers over time, facilities that consistently report data might differ from those with fluctuating data quality and completeness; therefore, data and results are not completely generalizable. Finally, trends observed after implementation of the new diagnosis code should be interpreted with caution, because higher proportions of CHS-involved ED visits might reflect increasing code adoption and improved recognition rather than true increases in CHS incidence.

### Implications for Public Health Practice

These findings highlight the need for increased awareness of CHS among clinicians and the public, but further educational efforts might be needed to prevent the cause of CHS: cannabis use. They also emphasize the importance of implementing evidence-based strategies to prevent cannabis use initiation and mitigate the consequences of cannabis use.

The observed higher proportions of CHS-involved ED visits after the new code was implemented in October 2025 potentially more accurately reflects true impact than previously observed proportions, underscoring the need for preparedness in emergency care settings, including appropriate clinical recognition and management of CHS. Clinicians can consider CHS in the differential diagnosis for patients with nausea, vomiting, and abdominal pain and can routinely assess cannabis use, including frequency, duration, product type, and route of use. Screening for substance use disorders and comorbid psychiatric conditions might also be warranted among patients reporting regular cannabis use (*10*).

Educational materials and communications campaigns should convey that younger age groups had high proportions of ED visits with CHS identification and that CHS might occur after shorter durations of cannabis use than was previously recognized (*7*,*8*). Expanded and tailored messaging might also be needed to raise awareness of CHS and other potential adverse health effects of cannabis use, particularly among younger populations. Communities can identify a wide range of effective substance use prevention and intervention strategies using CDC’s ENGAGE: Evidence-Based Strategies to Prevent Youth Substance Use resource for action.

Continued surveillance is important to better understand CHS epidemiology, including the potential role of high-potency THC and hemp-derived products and information on cannabis product characteristics (e.g., potency, type, or route of use) and to observe trends after code implementation to better assess prevalence. Monitoring trends across demographic groups and geographic areas can help identify populations at greatest risk and guide tailored prevention and intervention strategies.

## Footnotes

* Two data quality metrics were used: 1) coefficient of variation ≤45% and 2) weekly average discharge diagnosis informative ≥75. This method attenuates the extent of artificial increases when EDs newly report data to surveillance or when the data quality of the diagnosis variable improves.

† The remaining categories included American Indian or Alaska Native; Asian; Black or African American; Native Hawaiian or Pacific Islander; White; and multiple or other races. For analysis, Asian and Native Hawaiian or Pacific Islander were combined into a single category due to low case count.

§ Proportions of CHS-involved ED visits were calculated by summing the total number of visits during October 2025–May 2026, dividing by the total number of all-cause ED visits during October 2025–May 2026, and scaling to 10,000 ED visits.

¶ 45 C.F.R. part 46.102(l)(2), 21 C.F.R. part 56; 42 U.S.C. Sect. 241(d); 5 U.S.C. Sect. 552a; 44 U.S.C. Sect. 3501 et seq.

** Dabbing is the inhalation of highly concentrated tetrahydrocannabinol-based oils that are often heated using a blowtorch.
